# Systemic lupus erythematosus and pregnancy: clinical evolution, maternal and perinatal outcomes and placental findings

**DOI:** 10.1590/S1516-31802007000200005

**Published:** 2007-03-04

**Authors:** Fernanda Garanhani de Castro Surita, Mary Ângela Parpinelli, Ema Yonehara, Fabiana Krupa, José Guilherme Cecatti

**Keywords:** Lupus, Pregnancy, Perinatology, Placenta, Maternal welfare, Lupus, Gestação, Perinatologia, Placenta, Saúde materna

## Abstract

**CONTEXT AND OBJECTIVE:**

Systemic lupus erythematosus is a chronic disease that is more frequent in women of reproductive age. The relationship between lupus and pregnancy is problematic: maternal and fetal outcomes are worse than in the general population, and the management of flare-ups is difficult during this period. The aim here was to compare the outcomes of 76 pregnancies in 67 women with lupus, according to the occurrence or absence of flare-ups.

**DESIGN AND SETTING:**

An observational cohort clinical study evaluating the evolution of pregnant women with lupus who were receiving care at the prenatal outpatient clinic, Centro de Atenção Integral à Saúde da Mulher, Universidade Estadual de Campinas (CAISM/Unicamp), between 1995 and 2002.

**METHODS:**

Data were collected on a precoded form. The women were divided into two groups according to the occurrence or absence of flare-ups, as defined by the systemic lupus erythematosus disease activity index (SLEDAI). The presence or absence of flare-ups and renal involvement was considered to be the independent variable and the other results were dependent variables.

**RESULTS:**

Flare-ups occurred in 85.3% of cases, and were most significant when there was renal involvement. This was related to greater numbers of women with preeclampsia and poor perinatal outcome. Intrauterine growth restriction was more common in the women with active disease. Placental weight was significantly lower in the women with renal involvement.

**CONCLUSIONS:**

Flare-ups and renal involvement in lupus patients during pregnancy are associated with increased maternal and perinatal complications.

## RESUMO

**CONTEXTO E OBJETIVO:**

O lúpus eritematoso sistêmico é uma doença crônica que acomete preferencialmente mulheres em idade reprodutiva. A associação entre lúpus e gravidez é problemática e os resultados maternos e perinatais são piores que na população geral. O objetivo foi determinar os resultados de 76 gestações de 67 mulheres lúpicas segundo a atividade da doença.

**TIPO DE ESTUDO E LOCAL:**

Estudo clínico descritivo avaliando a evolução de gestantes lúpicas seguidas no Ambulatório de Pré-Natal Especializado do Centro de Atenção Integral à Saúde da Mulher, Universidade Estadual de Campinas (CAISM/Unicamp), no período de 1995 a 2002.

**MÉTODOS:**

Os dados foram coletados a partir de uma ficha pré-codificada. As mulheres foram divididas em dois grupos segundo atividade do lúpus eritematoso sistêmico (LES) na gestação, conforme o índice de atividade de doença lúpica SLEDAI (Systemic Lupus Erythematosus Disease Activity Index). A presença ou não de atividade de doença e de envolvimento renal foram consideradas variáveis independentes e os demais resultados as variáveis dependentes.

**RESULTADOS:**

A doença em atividade durante a gestação ocorreu em 85,3% dos casos, sendo o acometimento renal o mais importante, relacionando-se a um maior número de mulheres que tiveram pré-eclâmpsia e pior evolução perinatal. Restrição do crescimento intra-uterino foi mais freqüente nas mulheres com doença ativa. O peso da placenta também foi significativamente menor nas mulheres com envolvimento renal.

**CONCLUSÕES:**

A atividade da doença e o envolvimento renal do LES na gestação associam-se com o aumento de complicações maternas e perinatais.

## INTRODUCTION

Systemic lupus erythematosus (SLE) is an autoimmune disease of unknown etiology that can affect various organs and systems. Since it predominantly affects women (in the proportions of 9 to 1) and since in the majority of cases it is diagnosed between the ages of 20 and 40, it is the connective tissue disease that is most frequently associated with pregnancy and the puerperium. Remission of the disease around the time of conception is related to favorable pregnancy outcome.^1,2^ On the other hand, a diagnosis of SLE during pregnancy and flare-up around the time of conception or during pregnancy are related to poor prognosis for both the pregnancy and the course of the disease.^1^

Abortion, intrauterine growth restriction, prematurity, and perinatal morbidity and mortality are among the most common adverse perinatal outcomes in pregnant lupus patients. Flare-ups, nephritis and arterial hypertension are factors that increase the risk of perinatal complications,^2,3^ as is an association with antiphospholipid antibody syndrome (APS), which is present in 30-40% of SLE cases.^4^ Although rare, neonatal lupus is also a complication that may be serious and should always be considered in this population.

The adverse perinatal outcomes resul-ting from SLE are believed to occur as a consequence of immunological alterations in the placenta. The histology of the placenta frequently reveals vascular abnormalities in the uteroplacenta or alterations in coagulation. These lesions are generally similar to those found in preeclampsia, hypertension and diabetes mellitus.^1,5,6^ Improvements in the treatment and control of systemic lupus erythematosus have led to better quality of life for patients with this pathological condition and a consequent increase in the number of pregnancies in this population.^7^

## OBJECTIVE

The objective of the present study was to evaluate the clinical evolution, perinatal outcomes and most frequently observed placental alterations among pregnant lupus patients, according to the presence or absence of disease flare-ups. These women were receiving care at a specialized prenatal clinic, Centro de Atenção Integral à Saúde da Mulher (Women’s Full Healthcare Clinic), Universidade Estadual de Campinas (CAISM/Unicamp), over an eight-year period, and they gave birth at the same institution.

## METHODS

This observational cohort study was carried out at the specialized prenatal clinic CAISM/Unicamp, which is a tertiary clinic for high-risk pregnancies. The patients included in the study were followed up according to a specific protocol for the care of pregnant women with lupus, including investigation of the current clinical and laboratory conditions (cardiac, immunological, renal, hematological and hepatic status). They delivered their babies at the same institution between 1995 and 2002. A total of 67 women and 76 pregnancies were included in the study. They represent all of the cases with SLE that were managed at this service during this period. There was no loss to follow-up.

Data were collected on a precoded form and data entry was performed using the Epi-Info software program, version 6.1. After evaluating the general characteristics of the sample, the women were divided into two groups according to the presence or absence of flare-ups of the disease during pregnancy, as defined by the systemic lupus erythematosus disease activity index (SLEDAI).^8^ They were also divided into two groups according to whether renal involvement with SLE was detected or not.

Statistical analysis was carried out after performing consistency tests. Qualitative variables were analyzed using the χ² test and, when applicable, Fisher’s exact test. Student’s t test was used for comparison of the means of continuous quantitative variables. Statistical significance was esta-blished as p < 0.05. The study obtained prior approval from the institution’s Research Ethics Committee.

## RESULTS

During the study period, there were 23,676 deliveries at the institution, of which 76 occurred among lupus patients. Therefore, the rate of deliveries to lupus patients at this institution was 3.21:1000. The mean age of the lupus patients was 25.9 years (range: 18-39 years), and these patients had had between one and seven pregnancies, including the current one. Around 22.4% of the women had previously miscarried. The women in this study had attended a mean of 8.5 prenatal consultations. The mean time elapsed between diagnosis of lupus and commencement of pregnancy was 48.9 months, i.e. four to five years (Table 1).

**Table 1:** General characteristics and history of pregnant lupus patients. Campinas, 1995-2002

Maternal variables	Mean (n = 76)	SD
Age (years)	25.9	5.64
Number of prenatal visits	8.5	4.02
Disease duration (months)	48.9	39.0
	n	%
With history of abortion	17	22.4
First pregnancy	21	27.6
Nulliparous	28	36.8
Without living children	31	40.8
With history of cesarean sections	30	39.5

SD = standard deviation.

Of the 76 cases studied, 47 (61.8%) fulfilled the diagnostic criteria established by the American College of Rheumatology (ACR), while 17 (22.4%) had probable lupus (three positive criteria) and 12 (15.8%) had possible lupus (two positive criteria). The ACR criteria most frequently found in this population were: immunological disorders, blood abnormalities and renal involvement, serositis, butterfly-shaped rash and a positive antinuclear antibody (ANA) test (Table 2).

**Table 2:** Frequency of diagnostic criteria (American College of Rheumatology) among 76 pregnant lupus patients. Campinas, 1995-2002

Criterion	n	%
Immunological disorder	56	73.7
Hematological disorder	49	64.5
Renal disorder	47	61.8
Antinuclear antibody	46	60.5
Serositis	38	50.0
Butterfly wing rash	33	43.4
Discoid lupus	23	30.3
Photosensitivity	15	19.7
Arthritis	14	18.4
Mouth ulcer	6	7.9
Neurological disorder	5	5.7

Although the SLEDAI criteria do not recommend any specific test for evalua-ting the immunological profile, the ANA test was positive in 73% of cases during prenatal follow-up. There were positive findings of anti-DNA antibodies in 19%, anti-Ro in 37% and anti-La in 11% of the cases. Anticardiolipin antibodies (via immunoglobulin G and immunoglobulin M determination) and/or lupus anticoagulant (via Russell viper venom time and kaolin clotting time) were detected in 36% of the patients. Immunosuppressor treatment for SLE was required in 93.4% of the pregnant women. The immunosuppressors used included prednisone (at doses of 5 to 80 mg/day) and azathioprine. Antihypertensive drugs were used by 26.7% and aspirin and/or heparin by 14% of the women (data not presented in table).

Of the 67 women enrolled in this study, 20 (26.3%) suffered some form of hypertensive syndrome during pregnancy. Nine women (11.8%) had preeclampsia (defined as the raising of blood pressure after the twentieth week of pregnancy plus proteinuria above 300 mg over a 24-hour period), and all of these patients had some degree of renal involvement. Another three patients (4%) had gestational hypertension and eight (10.5%) had chronic hypertension. Some degree of lupus nephropathy was present in 32 women (45.1%) and there was no statistically significant difference in gestational loss between these patients and the women who had no renal alterations. However, the incidence of preeclampsia was greater, and the newborn infant’s weight and placental weight were lower in this group of women.

One maternal death occurred during the puerperium. This was a patient who had secondary pulmonary hypertension associated with lupus and who had had a flare-up during a twin pregnancy. She went into premature labor and underwent cesarean section at 28 weeks due to breech presentation of the first twin and premature labor. She developed central nervous system vasculitis and died from reentrant convulsions on the third day of the puerperium.

With regard to the type of delivery, 68.6% of the patients underwent cesarean section. The principal indication for this was fetal distress (32 cases). Of the 76 cases studied, 14 led to gestational loss. Of these, six were miscarriages (7.8%), three were cases of fetal death (3.9%) and five were neonatal deaths (6.5%) (Table 3). In all the cases in which the woman was not a primigravida, she had a history of at least one previous miscarriage. Among the cases of fetal death, all the patients had some degree of renal involvement.

**Table 3:** Type of delivery, indication for cesarean section and perinatal outcome among pregnant lupus patients. Campinas, 1995-2002

Type of delivery	n (70)*	%
Vaginal	22	31.4
Cesarean section	48	68.6
Fetal distress	32	66.6
Breech presentation	3	6.3
Repeated cesarean section	2	4.2
Other indications	11	22.9
**Perinatal morbidity-mortality**	**(n = 77)**	
Gestational loss rate (%)	14	18.2
Abortion	6	7.8
Fetal death	3	3.9
Neonatal death	5	6.5
Living	63	81.8
Weight: AGA	49	77.7
Weight: SGA	14	22.3
Prematurity rate	34	50.0
	**mean**	**SD**
Weight of newborn (g)	2324.35	866.57

AGA = adequate for gestational age; SGA = small for gestational age; SD = standard deviation.

* 6 cases of abortion were excluded.

There was one twin pregnancy; the rate of prematurity, as defined by the Capurro method, was 50.0% of all live births. The percentages of infants with Apgar score < 7 at the first and fifth minutes were 13.4% and 3.0%, respectively. The mean weight of the newborns was 2,324.35 grams. The majority were considered to be adequate for gestational age (AGA), while around 22.3% were considered small for gestational age (Table 3). Among the adverse neonatal outcomes occurring in the surviving infants, the most frequent were intraventricular hemorrhage (four cases), patent ductus arteriosus (five cases) and congenital dislocation of the hip (three cases). There were also two cases of thrombocytopenia, in which a diagnosis of neonatal lupus was made. There were no cases of atrioventricular blockage.

Histological examinations were carried out on 45 placentas and 44.4% of these were found to have no abnormalities. The mean placental weight was 403.36 ± 193.3 grams. The most common findings were infarct, edema and intervillous thrombosis.

**Figure 1:**
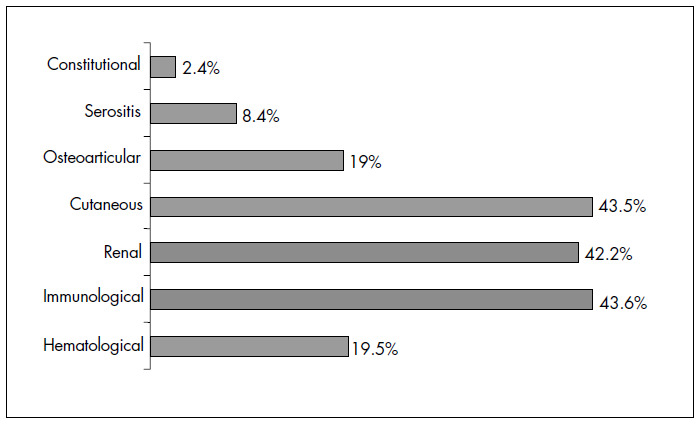
Disorder rates due to lupus activity among pregnant women. Campinas, 1995-2002.

The occurrence of disease flare-ups during pregnancy was defined as the presence of any of the SLEDAI criteria: hematological, immunological, renal, cutaneous, constitutional or osteoarticular alterations, or serositis. These alterations were analyzed in 68 of the 76 women in the study; 58 of them (85.3%) had some form of alteration (Figure 1). When the whole sample was dichotomized into those who did and those who did not have flare-ups according to the SLEDAI criteria, it was observed that preeclampsia only developed in those with some degree of active disease. The gestational loss rate, calculated by adding the percentages of miscarriages, fetal deaths and neonatal deaths, was not significantly different between the two groups. The women with disease flare-ups had a greater number of low-weight infants, although there was no difference in relation to prematurity or adequate weight for gestational age. There were no significant differences in the placental analysis, although a greater number of placentas with abnormalities were found in patients who had flare-ups (Table 4).

**Table 4:** Results according to systemic lupus erythematosus (SLE) flare-up and SLE renal involvement (nephropathy) among pregnant women. Campinas, 1995-2002

Results	Occurrence of flare-ups	No flare-ups	p	Renal involvement	No renal involvement	p
Preeclampsia	9/58	0/10	NS	9/32	0	< 0.05
Gestational loss rate	9/58	2/10	NS	2/32	2/39	NS
Low birth weight	32/52	2/9	< 0.05	20/27	14/36	< 0.05
Prematurity	29/52	4/9	NS	7/32	5/39	NS
Small for gestational age	14/52	0/9	NS	20/27	12/36	< 0.05
Placental characteristics	n = 38	n = 6		n = 9	n = 6	NS
Mean weight (g)	376.6	498	NS	330	459	< 0.05

NS = not significant.

On the other hand, when the group was dichotomized into patients who had and patients who did not have lupus nephropathy, statistically significant differences were seen in perinatal outcome. There was a greater number of low-weight infants, a greater rate of prematurity and fewer healthy infants at discharge from hospital among the patients with lupus nephropathy. Preeclampsia occurred exclusively in this group. The mean placental weight was also lower (Table 4). Women with antiphospholipid antibody syndrome were not significantly different with respect to gestational loss or placental abnormalities.

## DISCUSSION

Among woman with lupus, the interaction between the physiological changes to the immune system brought about by pregnancy and the pathological changes caused by the disease alter the normal course of the reproductive process.^4^ A higher rate of poor perinatal outcomes among women with systemic lupus erythematosus has been well-documented in the literature, particularly in women who have disease flare-ups during pregnancy, as was the case in the present series. In the literature, the flare-up rates during pregnancy range from 13 to 60%. This wide range may be explained by the absence of a universal standard for diagnosing lupus flare-ups, which is done subjectively in clinical practice.

Various indices have been created in an attempt to standardize the criteria that define flare-ups of this disease.^8^ These indices do not focus specifically on pregnant lupus patients and this makes their application in pregnancy difficult, since the clinical and laboratory standards of normality vary during this phase. However, in the absence of an adequate index for assessing pregnant women, and since the study demanded a non-subjective standard for defining disease flare-ups, we opted to use one of these existing indexes: the systemic lupus erythematosus disease activity index (SLEDAI).^8^ On the basis of this index, we defined groups of patients in whom the disease flared up or did not, in order to compare variables. In this study sample, flare-ups occurred in 85.3% of cases. This large difference in the numbers of patients with and without flare-ups, in comparison with other studies may be indicative of a lack of adequate contraceptive counseling in this population. In view of the importance of not having disease flare-ups around the time of a planned conception, this may have been a factor associated with the poor maternal and perinatal outcomes.

In the present study, not all the patients fulfilled at least four of the ACR criteria, which is the minimum requirement for reaching a confirmed diagnosis of lupus. However, these patients were managed in the same way as those with confirmed diagnosis, because of the strongly suggestive signs and symptoms presented and because SLE is an evolutive disease in which there may be a limited range of symptoms in its initial phase. On the other hand, careful prenatal care for these women may reduce the gestational risks and poor perinatal outcomes.

With regard to maternal complications, preeclampsia occurred only in patients who had flare-ups, and particularly in those with renal involvement. The incidence of prematurity was higher in this group of patients, which leads us to conclude that patients with maternal complications related to lupus flare-ups present higher perinatal risk. Differentiation between lupus flare-ups and preeclampsia is difficult in clinical practice. Preeclampsia occurs in approximately 13% of patients with lupus and is frequently confused with lupus nephritis.^4^ Some clinical and laboratory abnormalities are similar in these two conditions, such as edema, arterial hypertension, thrombocytopenia and other alterations in blood coagulation, and proteinuria. In addition, growth disorders and fetal vitality disorders may occur.

The laboratory test findings that may help in reaching a differential diagnosis are: abnormal urinary sedimentation with the presence of erythrocyte dysmorphism and cell casts, which are present in lupus nephritis, whereas preeclampsia is defined by the presence of isolated proteinuria; and hypocomplementemia and increased anti-DNA antibody titers, which are present in lupus nephritis and not in preeclampsia.^9,10^ Nevertheless, both conditions are serious and, while the treatment should be different in each case with respect to changing the immunosuppressors or increasing their doses if lupus flare-up is the first hypothesis, various complications such as changes in fetal vitality and maternal arterial hypertension should be treated similarly in both conditions.

The gestational loss rate, calculated from the sum of the percentages of miscarriages, fetal deaths and neonatal deaths, was 18.2%. In a previous study carried out in our center, in which 40 cases were analyzed over a period of nine years, this rate was 30%. This possibly demonstrates that some improvement has occurred in the care provided for pregnant lupus patients over recent years.^11^ However, this current rate is still much higher than what is found in normal pregnancies, albeit compatible with data in the literature from studies carried out among lupus patients.^12^

In the literature, a greater number of cases of miscarriage have been reported associated with antiphospholipid antibody syndrome. However, in our study, there was no statistically significant difference in the number of miscarriages between the two groups, perhaps due to the restricted sample size. The presence of APS was also not associated with gestational loss. It should be emphasized that, in all cases of fetal death, there was some degree of lupus renal involvement. These results are in agreement with reports in the literature, thus suggesting that lupus nephritis is predictive of poor prognosis for pregnancy.^13^

Pregnant SLE patients are normally referred for normal labor and delivery. However, the repercussions of their condition on the placenta and fetus, particularly if anticardiolipin antibody is present, increase the incidence of chronic or acute fetal distress and consequently increase the cesarean section rate among these women.^11^

The prematurity rate was high, reaching 74.1% among those with renal involvement, and this resulted in poor gestational prognosis, even among the patients whose pregnancy resulted in a live conceptus. In addition, prematurity in itself defines a greater degree of severity of neonatal morbidity, thus making the perinatal prognosis for such pregnancies even poorer.

The small-for-gestational-age rate (22.3%) was also higher than in normal populations, and it is important to stress the fact that in all these cases the mothers had disease flare-ups during pregnancy. In the literature, reports describe better prognosis for pregnancies in which no flare-ups occur, and recommend pregnancy only after a one-year period without disease flare-ups.^1,2^ In such cases, the aggression to the placenta is expected to be less, thus allowing adequate intrauterine development.

With regard to histology, although not all the placentas were analyzed, and despite the fact that the analyses were not specifically carried out for the purposes of this study, a greater rate of abnormalities was found in the placentas of this study population, particularly concerning the proportion of infarcted areas (more than 10%). Nevertheless, a comparative study with normal placentas is required, to evaluate the importance of these abnormalities. Immunohistochemistry would provide further important data for analyzing the physiopathology of the disease.

## CONCLUSIONS

There was a statistically significant difference with regard to the rate of low-weight newborn infants, between patients who had lupus flare-ups during pregnancy and those who did not. SLE patients with renal involvement during pregnancy had significantly greater rates of preeclampsia, prematurity and low-weight newborn infants. They also had smaller placentas than did the other patients with lupus. The presence of antiphospholipid antibodies was not related to poorer perinatal outcomes in this study sample.
